# A Study of the Fluid Intake, Hydration Status, and Health Effects among Pregnant Women in Their Second Trimester in China: A Cross-Sectional Study

**DOI:** 10.3390/nu15071739

**Published:** 2023-04-02

**Authors:** Yongye Song, Fan Zhang, Guotian Lin, Xing Wang, Limin He, Yanchuan Li, Yufei Zhai, Na Zhang, Guansheng Ma

**Affiliations:** 1 Department of Nutrition and Food Hygiene, School of Public Health, Peking University, 38 Xue Yuan Road, Haidian District, Beijing 100191, China; songyongye@bjmu.edu.cn (Y.S.); mags@bjmu.edu.cn (G.M.); 2 International School of Public Health and One Health, Hainan Medical University, 3 Xue Yuan Road, Longhua District, Haikou 571199, China; 3 Laboratory of Toxicological Research and Risk Assessment for Food Safety, Peking University, 38 Xue Yuan Road, Haidian District, Beijing 100191, China

**Keywords:** fluid intake, hydration status, health effects, pregnancy, amniotic fluid index

## Abstract

The fluid intake and hydration status during pregnancy may influence the health outcomes of both the mother and the fetus. However, there are few studies related to this. The aim of the present study was to investigate fluid intake behaviors among pregnant women in their second trimester, to evaluate their hydration status and pregnancy complications, and to further explore the association of fluid intake and the amniotic fluid index (AFI). Participants’ total fluid intake (TFI) levels were determined using a 7-day 24 h fluid intake questionnaire. The levels of water intake from food were not recorded or measured. Morning urine samples were collected, and both urine osmolality levels and urine specific gravity (USG) were tested to evaluate their hydration status. Fasting blood samples were also collected and measured for osmolality and complete blood count (CBC). A total of 324 participants completed the study. They were divided into four groups based on quartiles of TFI, including participants with lower (LFI_1_ and LFI_2_) and higher (HFI_1_ and HFI_2_) fluid intake levels. The median TFI was 1485 mL, and the median values of the four groups with different TFI levels were 1348, 1449, 1530, and 1609 mL, respectively. Only 3.4% of the participants attained the recommended value following an adequate water intake (1.7 L) level for pregnant women in China. Plain water was the main TFI resource (78.8~100.00%), and differences in the plain water intake levels among the four groups were evident (χ^2^ = 222.027, *p* < 0.05). The urine osmolality decreased sequentially with increasing TFI values from the LFI_1_ to HFI_2_ group, and significant differences in the urine osmolality levels among the four groups were evident (*p* < 0.05). Meanwhile, the percentage of dehydrated participants decreased from 26.8% in the LFI_1_ group to 0.0% in the HFI_2_ group (χ^2^ = 131.241, *p* < 0.05). Participants with higher TFI values had higher AFI values (χ^2^ = 58.386, all *p* < 0.05), and moderate-intensity correlations were found between TFI and urine osmolality, hydration status, and AFI (all *p* < 0.05). A large proportion of the participants had insufficient TFIs during the second trimester of pregnancy, and a proportion of the participants were dehydrated. The preliminary analysis showed that the AFI was correlated with the TFI during the second trimester of pregnancy. A sufficient TFI is necessary for pregnant women to improve their hydration status and may have effects on their health. The results can provide appropriate scientific references for the development of beneficial recommendations concerning adequate water intake levels for pregnant women in China.

## 1. Introduction

Water is an essential component of the organs and tissues of body. It accounts for 60~70% of the human body’s mass and approximately 83% of the blood. Water plays a vital role in various physiological functions, such as the maintenance of cellular shape and the regulation of both metabolism and temperature [1,2,3]. There are three sources of water: fluid intake from water and beverages, water intake from food, and endogenous water [4]. The main water output channels are the release of urine through urine systems, sweat through the skin’s surface, breath through the respiratory system, and feces through the digestive system [4,5]. Extensive evidence in the literature has determined that dehydration has an extensive impact, degrading one’s physical activity level [6], cognitive performance [7], gastrointestinal function [8], kidney excretion [9,10], and nervous system [11,12]. It can also lead to heat-related illnesses [13,14,15].

Pregnancy is a physiological stage experienced by women, where the body undergoes a series of physiological changes to meet the requirements for good fetal growth and development. During this period, an individual’s water balance level is altered due to the increase in total body water content [16]. The blood volume gradually increases from 6~8 weeks of pregnancy, attaining a peak value at 32~34 weeks, with an increase of 30~45%. As a result, the total blood volume increases by 1200~1800 mL compared to the pre-pregnancy period [17]. During the gestation stage, plasma volume increases and numerous changes occur in the urinary system, which lead to a significant increase in the requirement for water during pregnancy [18]. The kidneys of pregnant women become slightly enlarged, leading to an increased renal plasma flow (RPF) and glomerular filtration rate (GFR), as well as an increased excretion of metabolites, such as urea and creatinine [19,20]. It can be observed that changes also occur in the respiratory system of women during pregnancy, as ventilation rates increase by approximately 40% per minute and tidal volume increases by approximately 39% [21]. These lead to an increase in the amount of water expelled from the body through breathing. Adrenal and thyroid functions are relatively hyperactive during pregnancy, which speed up metabolism and increase water lost through perspiration. Women during pregnancy have an increased nutrient requirement that leads to an increased intake of food for appropriate energy and nutrition levels. Women during mid-pregnancy require an additional 300 kcal of energy per day, while women in the late-pregnancy stage require an additional 450 kcal of energy per day [22,23]. Water is the carrier of nutrient metabolism information, as it is essential for the digestion, absorption, circulation, and excretion of nutrients. Pregnancy demands higher levels of food intake and energy; as a result, more water is also required.

Hydration status is associated with pregnancy outcomes. Studies have shown that the incidence of chronic dehydration during pregnancy affects the weight and length of the child at birth, as well as its head and chest circumference [24]. It was also suggested in the literature that chronic hypovolemia resulting from insufficient water intake levels may be the main risk factor for the development of diabetes [25]. Gestational weight gain is estimated to be approximately 11 kg, a large portion of which is attributed to water retention. Of this, amniotic fluid accounts for approximately 800 g of this weight gain, blood accounts for approximately 1277 g, and extracellular fluid accounts for approximately 1496 g [26,27]. In addition, other studies have determined that a normal amniotic fluid volume is critical for normal fetal growth and development and this could be a predictor of fetal health [28]. Moreover, studies conducted in various countries suggested that a number of pregnant women presented insufficient fluid intake levels. Thus, there is an increased risk of dehydration during this critical period of the life cycle [16,29]. Insufficient fluid intake levels during pregnancy are believed to be associated with spontaneous abortions, preterm births, and fetal malformations [30]. Decreased amniotic fluid levels restrict fetal movement and increase the risk of greater cord pressure on the fetus. Consequently, the risk of hypoxia and fetal death is increased [31]. Although an adequate water intake level is important for both the mother and her fetus, the research conducted on the regulation of the total body water balance levels, daily water requirements, and biomarkers of hydration among pregnant and lactating women remains scarce. Even less is understood about how maternal hydration may impact either the mother or her fetus during pregnancy.

Previous studies have suggested that the changes in hydration status experienced during pregnancy influence amniotic fluid and pregnancy outcomes to some extent. A randomized controlled trial conducted in California on 40 pregnant women with a gestation period longer than 28 weeks divided the subjects into 2 groups at random. The intervention group (n = 20) was asked to drink 2000 mL of water within 2 h, while the control group was asked to drink 100 mL of water. The results showed that the amniotic fluid index (AFI) values for the intervention group after 2~5 h was significantly higher [32]. Additionally, a similar study was conducted on 84 gestational women with a gestational period longer than 35 weeks and with low AFI values. The result also showed that the oral intake of 2000 mL of water increased the AFI levels [33]. In a longitudinal cohort study, researchers measured the body composition of 440 pregnant women. They observed that the total body water level could be used to identify and determine the body’s edema status [34]. However, these studies mainly focused on the body water composition and health status of these women, rather than on their fluid intake. Studies conducted in various countries investigated the fluid intake behavior of male and female adults, and determined that both intake behaviors and volumes varied between different physiological stages and countries [35,36,37]. However, certain limitations are evident in the studies at present. Firstly, the data collected lack accuracy due to the lack of verified and authoritative scientific investigation methods available. Some studies investigated fluid intake levels among pregnant women; however, they did not analyze the health outcomes of participants with different fluid intake sources, volumes, and different hydration statuses [38,39]. Furthermore, the data do not provide sufficient detail concerning the health status of the tested individuals, as they are less likely to focus on the association between hydration levels and health effects experienced during pregnancy [31].

There are some countries that specify an appropriate water intake level for pregnant women. The recommended adequate water intake level for pregnant women by the American Medical Research Institute is 2700 mL/d. It is 300 mL/d higher than the level for adult women who are not pregnant. In Indonesia, women are advised to drink 240 mL more water during pregnancy than adult women who are not pregnant, which is 2080 mL/d. The Dietary Reference Intakes for Chinese Residents (2013) recommends that fluid intake levels during pregnancy should increase by 200 mL, on the basis of the recommendation for an adequate water intake level of 1.7 L for adult women [40]. The data concerning the fluid intake behaviors of pregnant women in China are few. For this reason, research data and recommendations obtained from other countries were used in the present study to develop recommendations for adequate water intake levels for pregnant women in China. However, these recommendations may be not applied well to pregnant women in China. Hence, an investigation of the fluid intake levels and the assessment of the health indicators of pregnant women in China is necessary.

In this study, the primary objective was to collect and assess the total fluid intake (TFI) levels as well as the fluid intake sources of women during the second trimester of pregnancy. The secondary objective was to assess urine and blood biomarkers to successfully evaluate pregnant women’s hydration status. Hence, these were used to analyze the relationship between TFI levels and the extant hydration indicators. Finally, the third objective was to explore the association between the AFI, pregnancy complications, and the TFI levels. The results of the study can provide scientific and useful references for the development of adequate water intake level recommendations for pregnant women living in China [31].

## 2. Methods

### 2.1. Sample Size Calculation

The incidence of new-onset hyperglycemia was used as a variable to calculate the sample size. A related study determined that the incidence of hyperglycemia in women during pregnancy was 0.16 [41]. In our study, the sample size was calculated using the following formula: n = t^2^p (1 − p)/e^2^. In the formula, “t” represents the corresponding statistic value when the confidence was set as 95%, that is, α = 0.05 and t = 1.96. In addition, “e” represents the error and was set as 4%. Considering a missed follow-up rate of 10%, 352 participants were required for the test.

### 2.2. Participants

A convenience sampling method was used to recruit pregnant women attending outpatient clinics at Haikou City Hospital, from August 2019 to March 2020, who met the inclusion criteria. Finally, 380 women in the mid-pregnancy stage were recruited for the study. The inclusion criteria were as follows: first pregnancy examination prior to the 13th week of gestation; an age range between 21 and 35 years; first pregnancy; a singleton pregnancy; and healthy. The exclusion criteria were as follows: smoker; habitual consumption of alcohol (>20 g/day) [42]; performs intensive physical activity; kidney, digestive system, or cardiovascular diseases; diabetes mellitus; or other diseases prior to the pregnancy.

### 2.3. Ethical Standards

The study protocol was reviewed and approved by the Ethical Review Committee of the Hainan Medical University. The ethical approval project identification code is 2018-4. The study was conducted according to the principles of the Declaration of Helsinki. Prior to the beginning of the study, all the participants read and voluntarily signed their informed consent forms.

### 2.4. Study Procedure

The cross-sectional study was conducted from August 2019 to May 2020. On the first day, the height, weight, and body composition of the participants were measured. From days 1 to 7, the 7-day 24 h fluid intake questionnaire was used, and fluid intake behaviors were recorded in real-time in free-living conditions [4]. Using a uniformly customized cup with a scale to the nearest 10 mL as a reference, the participants’ TFI values were evaluated and recorded. However, we did not measure or record the water intake level from food. Urine samples were collected in the morning on day 4 and were tested within 2 h of collection. Related urine biomarkers, including first-morning urine osmolality, USG, pH, urea, and concentration of creatinine, were measured by the researchers. On day 4, antecubital venous blood was collected to test for any related blood biomarkers, including blood glucose, blood lipid, hemoglobin, red blood cells, white blood cells, lymphocytes, and platelets. Moreover, pregnancy complications and other diseases or symptoms, including urinary tract infection, anemia, edema, and constipation, were assessed and diagnosed by obstetricians in accordance with the relevant physiological indicators. In addition, the temperature and humidity data concerning the location of the participants during the relevant days were measured and recorded in real time. The indicators collected during different study time points are presented in Table 1.

### 2.5. Measurement of Daily Total Fluid Intake (TFI)

The daily total water intake levels are the sum of the daily total fluid intake (TFI) and daily water intake levels from food. The TFI is the amount of fluid intake from water and beverages, excluding water from food. In this study, water intake levels from food were not recorded or measured.

Based on the questionnaire used in previous surveys conducted in China, the 7-day 24 h fluid intake record was revised according to the purpose of the survey [31,35]. Following standardized training by the investigators, the daily TFI levels of the participants were collected using a 7-day 24 h fluid intake record. The fluid intake level for each time period in the 7 consecutive days assessed was measured using a customized cup. The cup scale was accurate to 10 mL. The type of fluid intake was recorded in detail by the participants for each consecutive 7-day period, including plain water, dairy products, tea, sugar-sweetened beverages, and so on [43]. The types of plain water included in the study were tap, packaged, or mineral and purified. The types of dairy products we included were pure milk, yogurt, and other dairy products without the addition of sugar in the production process. Sugar-sweetened beverages (SSBs) included beverages with the addition of sugar in the beverage production process, including carbonated, fruit- and vegetable-juice, protein, coffee, plant-based, flavored, and special-purpose beverages. The types of tea we included were self-made green, scented, and black; however, we excluded tea beverages with added sugar from the study. The time and location of fluid intake activity were recorded by the participants. The records were photographed and sent by the participants to the investigators using a mobile phone, and were then reviewed every day by the investigators to ensure the accuracy and integrity of the fluid intake records.

### 2.6. Anthropometric Measurements

Height and weight: The participants were dressed in light clothing and stood barefoot on a height-and-weight scale with their backs facing a column, their torsos naturally straight, and their heads held upright throughout the process. The upper limbs were naturally lowered with the feet being positioned perpendicular to the ground. The investigator slowly moved the horizontal platen down the column to the participant’s head, and it was accurately read by the investigator with their eyes on the scale. The weight values were simultaneously recorded. The height and weight of the participants were measured twice by professional obstetricians using a height–weight meter (HDM-300; Huaju, Yiwu, China) following the standardized methods to the nearest 0.1 cm and 0.1 kg, respectively (BMI: weight (kg)/height squared (m)).

Blood pressure: Participants were asked to not perform any intense form of exercise prior to the blood pressure measurement and to sit still for 5 min. They were asked to sit on a chair with their body straight and relaxed. Blood pressure was measured twice by obstetricians to the nearest 2 mmHg using a desktop mercury sphygmomanometer (Yuwell, Danyang, China) with the left arm of the participant exposed, palm facing up, and a cuff wrapped around the left arm 1~2 cm above the inner elbow joint and secured in place. The systolic and diastolic blood pressures were determined according to the Korotkoff sound. Two measurements were obtained at 2 min intervals, and the average values of these two measurements were reported in this study.

### 2.7. Tests Conducted for Urine Biomarkers

Urine osmolality: The first-morning urine samples were collected in a sterile disposable urine sample cup. Using an osmotic pressure molar concentration meter (SMC 30C; Tianhe, Tianjin, China) with the freezing-point method, osmolality was determined by the laboratory physicians, which was then used to assess the hydration levels of the participants.

Urine specific gravity (USG): Using an automatic urinary sediment analyzer (FUS-200; Dirui; Changchun, China) with the uric dry-chemistry method, the urine samples were collected in sterile disposable urine sample cups to determine the USG by laboratory physicians.

Urine pH: Urine samples were tested within 2 h following their collection using an automatic urinary sediment analyzer (FUS-200; Dirui; Changchun, China) with the acid–base indicator method. The urine samples with different acidity and alkalinity levels produced different colors for the corresponding indicators, and the test results were recorded using the instrument.

Urea, urine creatinine, and uric acid: These factors were measured using the urease-glutamate dehydrogenase, sarcosine oxidase, and oxidative enzyme methods, respectively, using a fully automated biochemical analyzer (AU 5800, Beckman, Brea, CA, USA). The supernatant was collected for testing following centrifugation and the results were recorded by appropriate the instrument.

### 2.8. Judgement and Definition of Hydration Status

According to the urine osmolality, the participants were divided into three groups according to their hydration statuses as follows: a dehydrated status, normal hydration status, and optimal hydration status. A dehydrated status was defined as urine osmolality >800 mOsm/kg [44,45,46]. A normal hydration status was defined as urine osmolality ≤800 mOsm/kg but >500 mOsm/kg [47]. An optimal hydration status was defined as urine osmolality ≤500 mOsm/kg [48].

### 2.9. Determination of Blood Biomarkers

Blood osmolality: A volume of 2 mL fasting antecubital venous blood was collected from the patients in the morning and measured using an osmolality weight molar concentration tester (SMC 30C; Tianhe; Tianjin, China) in accordance with the Standard Operating Procedure for Determination of Osmolality Molar Concentration for Chinese Drug Testing Standard Practice 2016 Edition.

Blood glucose: Blood glucose levels were measured with an automatic biochemical analyzer (AU5800, Beckman, Brea, CA, USA) via spectrophotometry.

Other blood biomarkers: Blood was collected in vacuum tubes. An automatic biochemical analyzer (Cobas C 501; Roche; Basel, Switzerland) was used to detect the presence of hemoglobin, red blood cells, white blood cells, lymphocytes, and platelets.

### 2.10. Amniotic Fluid Index (AFI)

Amniotic fluid was measured using ultrasonography by professional medical technicians. Oligohydramnios was diagnosed by an AFI of <5 cm or maximum amniotic fluid pool depth of <2 cm [49].

### 2.11. Pregnancy Complications

Anemia: The hemoglobin of venous blood obtained from the elbow was tested using an automatic routine blood test device (MC600, Kubeier, Shenzhen, China) by the laboratory physicians. Anemia was defined as hemoglobin of <110 g/L, according to the criteria for anemia set by the World Health Organization and the United Nations International Children’s Emergency Fund (UNICEF) [50].

Hypocalcemia: The free-calcium concentration present in the blood samples was measured using an osmolality weight molar concentration meter (SMC 30C; Tianhe; Tianjin, China). Hypocalcemia was diagnosed as free calcium of <1.1 mmol/L [51].

Gestational diabetes mellitus (GDM): Blood glucose levels were determined from the venous blood obtained from the elbow of the participant using an osmotic pressure molar concentration meter (SMC 30C; Tianhe, Tianjin, China) by the laboratory physicians. GDM was diagnosed based on the results of the 100 g, 3 h oral glucose tolerance test (OGTT) conducted by the obstetricians. According to the recommendations of the Committee on Obstetric Practice, a definite diagnosis was made if two or more thresholds were met or exceeded by the patients. The thresholds of fasting blood glucose levels were as follows: fasting: 5.3 mmol/L, after 1 h: 10.0 mmol/L, after 2 h: 8.6 mmol/L, and after 3 h: 7.8 mmol/L [52].

Gestational hypertension: Hypertension during pregnancy was defined as systolic blood pressure levels of >140 mmHg and/or diastolic blood pressure levels of ≥90 mmHg sustained for two measurements [53].

Embolism: Platelets or indicators of coagulation function in the blood were measured using an osmolality weight molar concentration meter (SMC 30C; Tianhe; Tianjin, China). A diagnosis was made by specialist obstetricians according to the relevant criteria [54].

Intrauterine hypoxia: A diagnosis was made by specialist obstetricians according to the appropriate diagnostic criteria, in combination with the relevant physiological indicators [55].

Eclampsia and severe eclampsia: The laboratory physician used an automated biochemical analyzer (Cobas C501; Roche; Basel, Switzerland) to determine the urine protein levels. Related symptoms, including convulsions and/or unexplained comas experienced during or following pregnancy, were also monitored. Eclampsia and severe eclampsia were diagnosed by the obstetricians according to the relevant criteria [56].

Mild malnutrition: Participants’ height and weight were measured, and their BMI was also calculated. A BMI between 17.0 and 18.5 was diagnosed as a mild malnutrition status [57].

Other pregnancy complications: Diagnoses were made by specialist obstetricians or gynecologists with relevant physiological indicators according to the diagnostic criteria.

### 2.12. Temperature and Humidity in the Environment

Using a temperature and humidity meter (WSB-1-H2; Exasace; Zhejiang; China), the researchers measured and recorded the temperature and humidity of the participants’ living quarters at 9:00 a.m. and 3:00 p.m. every day during the study period. The accuracy levels of the temperature and humidity measurements were 1 °C and 0.1% RH, respectively.

### 2.13. Statistical Analysis

SPSS Statistics 26.0 (IBM Crop, Armok, NC, USA) was used to perform the statistical analysis. The data were subjected to normality tests. The results are reported as the mean ± standard deviation (SD) if the data were normally distributed. Otherwise, the median and quartile ranges (M and Q) are used to represent the data. Moreover, the data are presented as the mean number of participants, according to the diagnostic criteria. Differences in the normally distributed data (reported as mean ± SD), such as the urine osmolality, urine specific gravity (USG), urine pH, urea, urine creatinine, and uric acid, were compared using one-way ANOVA between the four groups. The Kruskal–Wallis H-test was used to compare the differences for non-normally distributed data (shown as M and Q) between the four groups. The chi-square test was used to compare the proportions of participants who met the adequate intake (AI) for China, fluid intake amounts, and percentages, hydration statuses, and pregnancy complications among the four groups. The differences evident between each of the two groups were compared using the Student–Newman–Keuls (SNK) method (*p* < 0.05). Spearman’s correlation coefficients were used to analyze the intensity of the correlations between fluid intake, hydration status, and AFI. Pearson’s correlation coefficients were used to analyze the intensity of the correlations between fluid intake and urine biomarkers. The significance level was set at 0.05 (*p* < 0.05).

## 3. Results

### 3.1. Participants’ Characteristics and the Environment

In this study, a total of 380 participants who met the inclusion were recruited. Among them, 28 participants dropped out voluntarily, 25 due to loss of contact and 3 due to incomplete data. Finally, 324 participants completed the study, resulting in an overall completion rate of 85.3%. The characteristics of these 324 participants are presented in Table 2.

The participants were divided into four groups, LFI_1_ (low fluid intake 1), LFI_2_ (low fluid intake 2), HFI_1_ (high fluid intake 1), and HFI_2_ (high fluid intake 2), according to the quartiles of total fluid intake (TFI) of the participants (Q1: 1211~1401 mL, Q2: 1402~1484 mL, Q3: 1485~1563 mL, Q4: 1564~1836 mL). The factors of age, height, weight, BMI, skeletal muscle, diastolic pressure, and systolic pressure did not significantly differ between the four groups (all *p* > 0.05).

The average temperature calculated was 27.6 ± 3.3 °C during this period. The average humidity was 78.3 ± 7.9% RH.

### 3.2. Measurement of Fluid Intake

Among the 324 participants, the median value for daily total fluid intake (TFI) was 1485 mL. The median values for the four groups with different TFI levels were 1348, 1449, 1530, and 1609 mL, respectively. The percentage of participants who met the adequate fluid intake level of China was 3.4%, based on a fluid intake of 1.7 L for pregnant women calculated on the basis of recommended water intake levels for women by the Chinese Nutrition Society [58]. In terms of the percentage of different sources presented in the TFI, the top-three sources were plain water, dairy products, and sugar-sweetened beverages (SSBs), which accounted for 93.5%, 4.1%, and 1.9% of the total sources, respectively.

There were statistically significant differences in the TFI values between the four groups (χ^2^ = 298.929, *p* < 0.05). The differences in the levels of plain water between the four groups were statistically significant (χ^2^ = 222.027, *p* < 0.05). The differences in the levels of SSBs between the four groups were statistically significant (χ^2^ = 22.236, *p* < 0.05). The percentage of SSBs in the TFI also significantly differed between the four groups (χ^2^ = 15.099, *p* < 0.05) (Table 3).

### 3.3. Measurement of Urine Biomarkers

Table 4 shows that, with the increase in the TFI, the osmolality of urine decreases from the LFI_1_ to HFI_2_ groups and is significantly different between all four groups (χ^2^ = 59.712, *p* < 0.05). The results show that there are statistically significant differences in the urine osmolality and urine specific gravity measurements between the participants with different TFI results (all *p* < 0.05). Approximately 26.5% of the participants had an optimal hydration status when evaluating the urine osmolality measurement. The proportion of dehydrated participants decreased from 26.8% in the LFI_1_ group to 0.0% in the HFI_2_ group (χ^2^ = 131.241, *p* < 0.05). The urine pH, urea, creatinine, and uric acid were not statistically significantly different between the four groups (Table 4).

### 3.4. Measurement of Blood Biomarkers

No statistically significant differences were observed in the concentration levels of blood glucose, blood lipids, white blood cell count, red blood cell count, hemoglobin, hematocrit, mean red blood cell volume, mean red blood cell hemoglobin content, platelet index, serum protein, serum bilirubin, and monocyte count between the participants in the four groups (all *p* > 0.05). The platelet volume distribution width and percentage of monocytes significantly differed between the four groups (χ^2^ = 7.847, *p* < 0.05; χ^2^ = 8.144, *p* < 0.05) (Table 5).

### 3.5. AFI, Pregnancy Complications, and Fluid Intake

When comparing the pregnancy complications evident in the second trimester of pregnancy among the participants with different TFIs, including anemia, hypocalcemia, gestational diabetes mellitus, hypertension, embolism, intrauterine hypoxia, severe eclampsia, and mild malnutrition, the results show that there are no statistically significant differences in the occurrence of pregnancy complications in the four groups. However, the AFIs significantly differ in the four groups (χ^2^ = 58.386, *p* < 0.05). The means ± standard deviations of AFIs for groups LFI_1_, LFI_2_, HFI_1_, and HFI_2_ are 10.81 (2.2), 11.37 (1.6), 12.44 (2.4), and 11.81 (2.5), respectively. From LFI_1_ to HFI_2_, the median values of the AFI in the four groups were 10.81, 11.37, 12.44, and 11.81, respectively. The AFI differed significantly in the four groups (χ^2^ = 58.386, *p* < 0.05) (Table 6). 

### 3.6. Correlations between Fluid Intake, Urine Blood Biomarkers, Hydration Status, and AFI

Moderate-intensity negative correlations were found between the TFI, plain water intake, and urine osmolality (*r* = −0.397, *p* < 0.05; *r* = −0.377, *p* < 0.05). In contrast, the correlations between the TFI and USG were low and negative (*r* = −0.111, *p* < 0.05). Moderate-intensity positive correlations were found between the TFI, plain water intake, and hydration status (*r* = 0.417, *p* < 0.05; *r* = 0.370, *p* < 0.05). Moderate-intensity positive correlations were also found between the TFI, plain water intake, and AFI (*r* = 0.437, *p* < 0.05; *r* = 0.418, *p* < 0.05) (Table 7).

## 4. Discussion

In this study, we investigated TFIs, hydration status, and related health indicators of women during the second trimester of pregnancy in China. Furthermore, we analyzed the differences in the AFIs and pregnancy complication cases with different TFIs. Simultaneously, the urine and blood biomarkers of the participants were measured to assess their hydration status. The results obtained in this study suggest that most pregnant women during their second trimester did not drink enough fluids. Additionally, different AFIs existed for pregnant women with different TFIs. In this study, the median daily TFI value of the participants was 1488 mL, which was lower than the 1500 mL recommended per day by the Chinese Dietary Guidelines (2022) for non-pregnant adult women and 1700 mL recommended per day for pregnant women. A total of 47% of the participants attained the recommended level of adequate fluid intake for Chinese adult women (1500 mL), and only 3% of the participants met the water intake requirement of 1700 mL per day for pregnant women [40]. A study conducted on women during the early pregnancy stage in China determined that the average daily fluid intake of 98 pregnant women was 1508 mL, which was also below the recommended amount of 1700 mL/d [59]. A survey conducted on 943 Chinese women in the mid-pregnancy stage concerning water intake levels showed that only 23% of pregnant women met the recommendations for adequate fluid intake levels [60]. An Indonesian study investigated the water intake levels of 300 women during pregnancy and determined that the total drinking fluid intake value was 2.3 L/d, and 42% of the participants’ daily total drinking fluid levels were below the recommended amount. Water intake requirements are related to several factors, including climate and dietary pattern. Since a discrepancy between climate, environmental, and dietary pattern factors exist between Indonesian and Chinese residents, the adequate intake level of water is higher in Indonesia than in China. Therefore, the water intake behavior of Chinese residents is different to that practiced in other countries, which is not comparable. It is necessary to conduct research on the water intake behavior of Chinese residents. In current studies, it can be observed that inadequate TFI during pregnancy is common among women in different countries.

The main result was that plain water was the main source of TFIs in all four groups, accounting for 78.8~100.00%. This was consistent with the results obtained for adults and children living in China [38,44,48,61,62,63,64]. Dairy products were the second-highest contributors among participants in all four groups, although the amount of milk (median: 64 mL) was considerably lower than the amount recommended by the Chinese Nutrition Society (300 mL). This result is consistent with that obtained by one previous study, which was conducted on male adults in China [64]. In our study, the hydration status of the four groups improved when the TFI was significantly increased (χ^2^ = 59.712, *p* < 0.05). Previous studies have shown that dehydration, caused by insufficient fluid intake levels, was common among individuals [65]. Another study conducted on male and female Chinese adults revealed that it was common to identify dehydrated participants [66]. In this study, water intake levels from food were not measured. In addition to fluids and beverages as intake sources, the human body also obtains water from food to maintain a normal hydration level. The proportion of water intake from food to total water intake (sum of water intake from food, fluid intake, and beverage intake) for residents in European and American countries is approximately 20%, but that of Chinese residents is approximately 50%. This outcome may be attributed to the different dietary patterns, cooking methods, and some other factors that exist in different countries. In Western countries, the diets of residents mainly involve the consumption of animals as a food source with a low water content, such as meat. In China, the diet of residents mainly involves the consumption of plants with a higher water content. The common cooking methods practiced in China, such as steaming, stewing, and stir-frying, not only retain better water levels in the ingredients, but also add additional water in the process of cooking. The common cooking methods used in the West are frying and baking. The use of high temperatures during these cooking processes can remove most of the water contained in the food. Analysis of water taken in from the consumption of food during pregnancy will provide further, important information; a lack of this provision is one of the limitations of this study.

The data presented in Table 4 suggest that, of the six biomarkers we tested, urine osmolality and urine specific gravity were the most sensitive to the TFI. Participants’ urine osmolality decreased as the TFI increased, which was similar to the results obtained by other studies [66,67]. With reference to the results of the previous studies, it can be observed that urine osmolality was more sensitive to hydration status than urine pH, urea, urine creatinine, and uric acid [68]. This suggests that it is reasonable to select urine osmolality as the best indicator of hydration status. The blood biomarkers did not change significantly with different TFIs in each group, since only the width of the platelet volume distribution and percentage of monocytes differed. This was in agreement with the results obtained by a study conducted in Beijing that determined that the urine and blood biomarkers of young men differed in groups with different fluid intake levels [61]. Several studies have also shown that blood indicators are good markers for assessing dehydration status in individuals; however, these were not sensitive to a mild hydration status [69].

The results of the study also revealed that different AFIs existed between pregnant women with different TFIs (*p* < 0.05). The AFI values were higher in the group with higher TFI values. Moderate-intensity correlations were found between the TFI, plain water intake, and AFI. The results suggested that fluid intake improved the hydration status and AFI. The results of a randomized controlled trial performed in California on 40 women after more than 28 weeks of gestation were similar to the results obtained in our study [32]. Additionally, the results are in line with a previous study conducted on 84 women with low AFIs, which revealed that the oral intake of 2 L of water increased AFI values [33]. However, the mechanism functioning between fluid intake or hydration status and changes in amniotic fluid is also unclear.

Our study presented some limitations. Firstly, we did not investigate the water levels obtained from the consumption of food. However, our previous research indicated that this was an important source of total water intake, accounting for 50.7 to 51.5% [64]. There may be differences in the water intake levels from food among participants with different TFIs; therefore, it is necessary to perform an in-depth approach to study the dietary intake type and level of participants to obtain the total water intake value. Secondly, only the AFI during the second trimester of pregnancy was investigated in this study. This resulted in a limitation considering the rapid increase in amniotic fluid volume in the third trimester. Hence, in additional studies, it is necessary to focus on the relationship between the TFI and AFI during the entire pregnancy and to investigate the pregnancy outcomes. Thirdly, this study is a cross-sectional rather than a longitudinal survey; however, differences are evident in the physiological status of the participants at different stages of their pregnancy. Thus, studies conducted on a higher number of participants during different gestational periods are necessary. It is also necessary to longitudinally monitor infants’ health status to observe the long-term effects of chronic maternal dehydration. The data collection was conducted during a specific time of the year, which means that seasonality was not considered in the study. However, Hainan is located at the southernmost point of China, on the northern edge of the tropics. Body fluid intake levels of Hainan inhabitants may be seasonally affected. In summary, the time-point data obtained from this cross-sectional survey may not be comprehensive.

## 5. Conclusions

The TFI was inadequate for pregnant women assessed during the second trimester, with only 11% of participants meeting the recommendation for adequate intake of water for pregnant women living in China. The participants presented a poor hydration status. Participants with lower fluid intake levels had lower AFIs. It is recommended that further attention is paid to the cultivation of healthy water intake behavior, and adequate water intake is important for maintaining a normal hydration and health status. The results can provide a scientific reference for the development of good recommendations for appropriate water intake levels for pregnant women in China. Further studies with long-term dynamic monitoring of water intake levels and hydration status during the different stages of pregnancy are necessary to effectively analyze their impact on pregnancy outcomes.

## Figures and Tables

**Table 1:** The indicators and pregnancy outcomes collected at different time points in this study.

	Day 1	Day 2	Day 3	Day 4	Day 5	Day 6	Day 7
Individual information	√						
Anthropometric	√						
7-day 24 h fluid intake questionnaire	√	√	√	√	√	√	√
Blood biomarkers				√			
Morning urine and related biomarkers				√			
Environment	√	√	√	√	√	√	√

**Table 2:** Characteristics of participants.

	LFI_1_	LFI_2_	HFI_1_	HFI_2_	Total		
	(n = 82)	(n = 81)	(n = 81)	(n = 80)	(n = 324)	χ^2^	*p*
Age (years)	28.5 (4.0)	28.0 (6.0)	30.0 (6.0)	29.0 (6.0)	29.0 (6.0)	2.006	0.571
Height (cm)	156.5 (7.0)	157.0 (7.3)	156.0 (7.0)	156.0 (7.0)	156.8 (6.9)	2.374	0.498
Weight (kg)	54.2 (10.0)	53.4 (7.5)	53.2 (0.7)	53.5 (6.8)	53.5 (8.3)	1.908	0.375
BMI (kg/m^2^)	22.5 (3.3)	22.2 (3.4)	22.0 (4.0)	21.9 (2.5)	22.1 (3.0)	2.605	0.462
Skeletal muscle	40.2 (6.1)	39.7 (6.5)	40.7 (5.1)	40.2 (4.8)	40.2 (5.4)	1.152	0.683
Blood pressure							
Systolic (mmHg)	115 (11)	113 (10)	112 (10)	112 (15)	113 (12)	2.296	0.513
Diastolic (mmHg)	78 (9)	76 (10)	76 (10)	77 (8)	77 (9)	3.011	0.390

Note: Values represent medians (quartile ranges). The Kruskal–Wallis test was used to analyze the differences in the indices between participants in different groups. A *p*-value of less than 0.05 was considered significant. BMI: body mass index; LFI_1_: low fluid intake 1; LFI_2_: low fluid intake 2; HFI_1_: high fluid intake 1; HFI_2_: high fluid intake 2.

**Table 3:** Composition of fluid intake of participants with different TFI levels.

	LFI_1_	LFI_2_	HFI_1_	HFI_2_	Total		
	(n = 82)	(n = 81)	(n = 81)	(n = 80)	(n = 324)	χ^2^	*p*
Daily TFI (mL) *	1347.9 (65)	1448.6 (43)	1530.0 (41)	1609.3 (78)	1485 (163)	298.929	<0.001
Percentage meeting Chinese water AI level (%) ^#^	0 (0.0)	0 (0.0)	0 (0.0)	11 (13.8)	11 (3.4)	34.622	<0.001
TFI sources							
Plain water							
Amount (mL) *	1225.0 (87)	1354.3 (81)	1440.0 (86)	1507.9 (95)	1384.3 (172)	222.027	<0.001
Percent (%) ^#^	94.2 (4.8)	93.4 (5.6)	94.4 (5.5)	92.7 (4.7)	93.5 (5.1)	7.391	0.060
SSBs							
Amount (mL) *	0.0 (34)	28.6 (64)	31.4 (64)	52.1 (86)	59.3 (57)	22.236	<0.001
Percent (%) ^#^	0.0 (2.6)	1.9 (4.5)	2.0 (4.3)	3.2 (5.2)	1.9 (4.2)	15.099	0.002
Dairy products							
Amount (mL) *	58.6 (60)	57.1 (58)	58.6 (48)	68.6 (64)	28.6 (64)	4.820	0.185
Percent (%) ^#^	4.4 (4.5)	3.9 (4.1)	3.8 (3.1)	4.2 (4.00)	4.1 (3.7)	4.540	0.209
Tea							
Amount (mL) *	0.0 (0.0)	0.0 (0.0)	0.0 (0.0)	0.0 (0.0)	0.0 (0.0)	0.000	1.000
Percent (%) ^#^	0.0 (0.0)	0.0 (0.0)	0.0 (0.0)	0.0 (0.0)	0.0 (0.0)	0.000	1.000

Note: * Values represent medians (quartile ranges) and were compared using the Kruskal–Wallis test; ^#^ values represent n (percentage) and were compared using the chi-squared test. A *p*-value of less than 0.05 was considered significant. AI represents recommendations for adequate intake level. The AI recommendation for TFI levels for male adults set by the Chinese nutrition society is 1.7 L. TFI: total fluid intake; SSBs: sugar-sweetened beverages; LFI_1_: low fluid intake 1; LFI_2_: low fluid intake 2; HFI_1_: high fluid intake 1; HFI_2_: high fluid intake 2.

**Table 4:** Urine indexes for participants with different TFI levels.

	LFI_1_	LFI_2_	HFI_1_	HFI_2_	Total	F	χ^2^	*p*
	(n = 82)	(n = 81)	(n = 81)	(n = 80)	(n = 324)			
Urine osmolality (mOsm/kg) *	718.5 ± 143.2	678.9 ± 168.8	571.3 ± 204.8	530.9 ± 178.0	625.5 ± 190.3	20.623		<0.001
Hydration status								
Optimal hydration status (n,%) ^#^	6(7.3%)	13(16.1%)	31(38.3%)	36(45.0%)	86(26.5)			
Normal yhdration status (n,%) ^#^	54(65.9%)	59(72.8%)	44(54.3%)	44(55.0%)	201(62.1%)		131.241	<0.001
Dehydrated status (n,%) ^#^	22(26.8%)	9(11.1%)	6(7.4%)	0(0.0%)	37(11.4%)			
Urine specific gravity (USG) *	1.2 ± 0.0	1.0 ± 0.0	1.0 ± 0.0	1.0 ± 0.7	1.0 ± 0.0	3.093		0.027
Urine pH *	6.0 ± 0.8	6.1 ± 0.8	6.2 ± 0.7	6.2 ± 0.7	0.1 ± 0.7	2.110		0.099
Urea (mmol/L) *	3.9 ± 1.1	4.1 ± 1.0	4.0 ± 1.0	4.0 ± 0.9	4.0 ± 1.0	0.709		0.547
Urine creatinine (mmol/L) *	59.4 ± 10.4	57.5 ± 9.7	58.8 ± 10.9	57.4 ± 9.6	58.3 ± 10.2	0.771		0.511
Uric acid (UA) (mmol/L) *	260.8 ± 56.3	259.5 ± 55.5	261.3 ± 62.3	276.6 ± 69.8	264.5 ± 61.3	1.389		0.246

Note: * Values represent the mean ± standard deviation (SD) and were compared using one-way ANOVA; ^#^ values represent n (percentage) and were compared using the chi-squared test. A *p*-value of less than 0.05 was considered significant. LFI_1_: low fluid intake 1; LFI_2_: low fluid intake 2; HFI_1_: high fluid intake 1; HFI_2_: high fluid intake 2.

**Table 5:** Blood indexes of participants with different TFI levels.

	LFI_1_	LFI_2_	HFI_1_	HFI_2_	Total		
	(n = 82)	(n = 81)	(n = 81)	(n = 80)	(n = 324)	χ^2^	*p*
Blood glucose (mmol/L)	5.1 (0.7)	5.09 (0.7)	5.3 (0.6)	5.3 (0.8)	5.2 (0.6)	3.168	0.366
Blood lipid							
TG (mmol/L)	0.9 (0.7)	0.9 (0.5)	0.8 (0.5)	1.0 (0.5)	0.9 (0.5)	2.124	0.547
TC (mmol/L)	4.8 (0.9)	4.8 (1.0)	5.0 (1.5)	5.0 (1.0)	4.9 (1.1)	0.568	0.904
HDL (mmol/L)	1.7 (0.7)	1.7 (0.7)	1.7 (0.7)	1.8 (0.7)	1.7 (0.7)	1.074	0.783
LDL (mmol/L)	2.6 (0.9)	2.7 (0.8)	2.9 (1.1)	2.8 (0.9)	2.8 (0.9)	1.648	0.649
Leukocyte count (10^9^/L)	6.5 (2.1)	6.2 (2.2)	6.3 (2.1)	6.0 (2.2)	6.2 (2.1)	1.218	0.749
Red blood cell count (10^12^/L)	4.6 (0.5)	4.7 (0.6)	4.7 (0.5)	4.6 (0.5)	4.7 (2.1)	0.640	0.887
Hemoglobin concentration (g/L)	132.0 (2.0)	133.0 (13.0)	134.0 (13.0)	130.0 (12.0)	132.0 (13.8)	1.407	0.704
Hematocrit	40.4 (4.2)	40.3 (3.4)	40.8 (3.6)	40.2 (4.0)	40.5 (0.5)	0.416	0.937
Mean red blood cell volume (fL)	87.0 (7.1)	88.8 (8.5)	86.3 (7.1)	88.2 (9.8)	87.8 (8.3)	0.387	0.943
Mean hemoglobin content (pg)	326.5 (18.3)	328.0 (15.0)	326.0 (17.0)	324.0 (15.5)	326.0 (16.0)	1.461	0.691
Platelet count (10^9^/L)	299.5 (87.0)	300.0 (93.0)	302.0 (72.0)	287.0 (93.0)	299.0 (86.5)	0.961	0.811
Mean platelet volume (fL)	10.2 (1.0)	10.2 (1.1)	10.2 (1.0)	10.2 (1.0)	10.2 (1.0)	1.677	0.642
Platelet volume distribution width	11.4 (1.8)	11.4 (3.2)	12.1 (5.0)	12.1 (3.8)	11.7 (3.0)	7.847	0.049
Platelet pressure	0.3 (0.1)	0.3 (0.1)	0.3 (0.1)	0.3 (0.1)	0.3 (0.1)	0.910	0.823
Total serum protein (g/L)	74.7 (4.1)	75.1 (4.0)	75.1 (4.7)	75.4 (4.7)	75.1 (4.3)	1.075	0.783
Total serum bilirubin (μmoI/L)	10.0 (5.1)	9.7 (4.1)	9.4 (3.4)	8.4 (4.1)	9.3 (4.4)	5.668	0.129
Direct bilirubin (μmoI/L)	2.3 (1.5)	2.3 (1.1)	2.3 (0.9)	2.2 (0.9)	2.3 (1.1)	1.649	0.648
Percentage of lymphocyte (%)	35.1 (9.6)	34.1 (9.9)	34.8 (10.5)	35.1 (8.3)	34.9 (9.6)	0.573	0.903
Percentage of monocyte (%)	5.4 (1.3)	5.4 (1.8)	5.3 (1.7)	6.1 (1.7)	5.5 (0.7)	8.144	0.043
Lymphocyte count (10^9^/L)	35.0 (9.6)	34.1(9.9)	34.8 (10.6)	35.0 (8.6)	34.8 (9.4)	0.540	0.910
Monocyte count (10^9^/L)	0.4 (0.1)	0.3 (0.1)	0.3 (0.1)	0.3 (0.2)	0.3 (0.1)	1.989	0.575

Note: Values represent medians (quartile ranges) and were compared using the Kruskal–Wallis test. A *p*-value of less than 0.05 was considered significant. TC: total cholesterol; TG: triglyceride; LDL: low-density lipoprotein; HDL: high-density lipoprotein; LFI_1_: low fluid intake 1; LFI_2_: low fluid intake 2; HFI_1_: high fluid intake 1; HFI_2_: high fluid intake 2.

**Table 6:** Pregnancy complications and AFIs for participants with different TFI levels.

	LFI_1_ (n = 82)		LFI_2_ (n = 81)		HFI_1_ (n = 81)		HFI_2_ (n = 80)			
	Negative	Positive	Negative	Positive	Negative	Positive	Negative	Positive	χ^2^	*p*
Anemia *	79 (24.4%)	3 (0.9%)	78 (24.1%)	3 (0.9%)	79 (24.4%)	2 (0.6%)	79 (24.4%)	1 (0.3%)	1.213	0.750
Hypocalcemia *	78 (24.1%)	4 (1.2%)	79 (24.4%)	2 (0.6%)	81 (25.0%)	0 (0.0%)	80 (24.7%)	0 (0.0%)	7.339	0.062
Gestational diabetes mellitus *	81 (25.0%)	1 (0.3%)	81 (25.0%)	0 (0.0%)	80 (24.7%)	1 (0.3%)	80 (24.7%)	0 (0.0%)	1.988	0.575
Hypertension *	80 (24.7%)	2 (0.6%)	79 (24.4%)	2 (0.6%)	81 (25.0%)	0 (0.0%)	79 (24.4%)	1 (0.3%)	2.205	0.531
Embolism *	81 (25.0%)	1 (0.3%)	81 (25.0%)	0 (0.0%)	81 (25.0%)	0 (0.0%)	80 (24.7%)	0 (0.0%)	2.960	0.398
Intrauterine hypoxia *	82 (25.3%)	0 (0.0%)	81 (25.0%)	0 (0.0%)	80 (24.7%)	1 (0.3%)	80 (24.7%)	0 (0.0%)	3.009	0.390
Severe eclampsia *	82 (25.3%)	0 (0.0%)	81 (25.0%)	0 (0.0%)	81 (25.0%)	0 (0.0%)	80 (24.7%)	0 (0.0%)	-	-
Mild malnutrition *	80 (24.7%)	2 (0.6%)	79 (24.4%)	2 (0.6%)	79 (24.4%)	2 (0.6%)	80 (24.7%)	0 (0.0%)	2.005	0.571

Note: * Values represent n (percentage) and were compared using the chi-squared test. For example, the percentage of patients with anemia in group LFI_1_ was calculated by dividing the number of participants with anemia in the group by the total participants included in the study. A *p*-value of less than 0.05 was considered significant. LFI_1_: low fluid intake 1; LFI_2_: low fluid intake 2; HFI_1_: high fluid intake 1; HFI_2_: high fluid intake 2.

**Table 7:** Correlations between fluid intake, urine biomarkers, hydration status, and AFI.

	Plain Water		SSBs		Dairy Products		TFI	
	*r*	*p*	*r*	*p*	*r*	*p*	*r*	*p*
Urine osmolality (mOsm/kg)	−0.377	<0.001	−0.072	0.199	0.005	0.932	−0.397	<0.001
Urine specific gravity (USG)	−0.089	0.110	0.001	0.980	−0.066	0.235	−0.111	0.046
Hydration status	0.370	<0.001	−0.071	0.200	−0.010	0.861	0.417	<0.001
AFI	0.418	<0.001	0.049	0.383	−0.013	0.822	0.437	<0.001

Note: Spearman’s correlation coefficients were used to analyze the intensity of the correlations between fluid intake, hydration status, and AFI. Pearson’s correlation coefficients were used to analyze the intensity of the correlations between fluid intake and urine biomarkers. A *p*-value of less than 0.05 was considered significant. SSBs: sugar-sweetened beverages; TFI: total fluid intake.

## Data Availability

The data are available from the corresponding authors upon reasonable request.

## Abbreviations

LFI	Low fluid intake
HFI	High fluid intake
TFI	Total fluid intake
AFI	Amniotic fluid index
SSB	Sugar-sweetened beverage

## References

[1] Jéquier E., Constant F. (2010). Water as an essential nutrient: The physiological basis of hydration. Eur. J. Clin. Nutr..

[2] Häussinger D. (1996). The role of cellular hydration in the regulation of cell function. Biochem. J..

[3] Goodlin R.C., Anderson J.C., Gallagher T.F. (1983). Relationship between amniotic fluid volume and maternal plasma volume expansion. Am. J. Obstet. Gynecol..

[4] Zhang N., Zhang F., Chen S., Han F., Lin G., Zhai Y., He H., Zhang J., Ma G. (2020). Associations between hydration state and pregnancy complications, maternal-infant outcomes: Protocol of a prospective observational cohort study. BMC Pregnancy Childbirth.

[5] Olivares M., Uauy R. (2005). Comprehensive Overview Paper: Essential Nutrients in Drinking Water.

[6] Cheuvront S.N., Kenefick R.W. (2014). Dehydration: Physiology, assessment, and performance effects. Compr. Physiol..

[7] Lieberman H.R. (2007). Hydration and cognition: A critical review and recommendations for future research. J. Am. Coll. Nutr..

[8] Arnaud M. (2003). Mild dehydration: A risk factor of constipation?. Eur. J. Clin. Nutr..

[9] Schoen E.J. (1957). Minimum urine total solute concentration in response to water loading in normal men. J. Appl. Physiol..

[10] Lindeman R.D., Van Buren H.C., Raisz L.G. (1960). Osmolar renal concentrating ability in healthy young men and hospitalized patients without renal disease. N. Engl. J. Med..

[11] Schroeder C., Bush V.E., Norcliffe L.J., Luft F.C., Tank J., Jordan J., Hainsworth R. (2002). Water drinking acutely improves orthostatic tolerance in healthy subjects. Circulation.

[12] Lu C., Diedrich A., Tung C., Paranjape S.Y., Harris P.A., Byrne D.W., Jordan J., Robertson D. (2003). Water ingestion as prophylaxis against syncope. Circulation.

[13] Casa D.J., DeMartini J.K., Bergeron M.F., Csillan D., Eichner E.R., Lopez R.M., Ferrara M.S., Miller K.C., O’Connor F., Sawka M.N. (2015). National athletic trainers’ association position statement: Exertional heat illnesses. J. Athl. Train..

[14] Adams W.M., Hosokawa Y., Casa D.J. (2016). Body-cooling paradigm in sport: Maximizing safety and performance during competition. J. Sport Rehabil..

[15] Medicine A.C.O.S., Armstrong L.E., Casa D.J., Millard-Stafford M., Moran D.S., Pyne S.W., Roberts W.O. (2007). American college of sports medicine position stand. Exertional heat illness during training and competition. Med. Sci. Sports Exerc..

[16] Bardosono S., Prasmusinto D., Hadiati D.R., Purwaka B.T., Morin C., Pohan R., Sunardi D., Chandra D.N., Guelinckx I. (2016). Fluid intake of pregnant and breastfeeding women in Indonesia: S cross-sectional survey with a seven-day fluid specific record. Nutrients.

[17] Vricella L.K. (2017). Emerging understanding and measurement of plasma volume expansion in pregnancy. Am. J. Clin. Nutr..

[18] Hyteen F.E., Paintin D.B. (1963). Increase in plasma volume during normal pregnancy. J. Obstet. Gynaecol. Br. Commonw..

[19] Lotan Y., Antonelli J., Jiménez I.B., Gharbi H., Herring R., Beaver A., Denni A., Von Merveldt D., Carter S., Cohen A. (2017). The kidney stone and increased water intake trial in steel workers: Results from a pilot study. Urolithiasis.

[20] Beers K., Patel N. (2020). Kidney Physiology in Pregnancy. Adv. Chronic Kidney Dis..

[21] Cornelis T., Odutayo A., Keunen J., Hladunewich M. (2011). The kidney in normal pregnancy and preeclampsia. Semin. Nephrol..

[22] Butte N.F., King J.C. (2005). Energy requirements during pregnancy and lactation. Public Health Nutr..

[23] Rugină C., Mărginean C.O., Meliţ L.E., Giga D.V., Modi V., Mărginean C. (2020). Relationships between excessive gestational weight gain and energy and macronutrient intake in pregnant women. J. Int. Med. Res..

[24] Mulyani E.Y., Hardinsyah, Briawan D., Santoso B.I., Jus’At I. (2021). Effect of dehydration during pregnancy on birth weight and length in West Jakarta. J. Nutr. Sci..

[25] Thornton S.N. (2014). Diabetes and hypertension, as well as obesity and Alzheimer’s disease, are linked to hypohydration-induced lower brain volume. Front. Aging Neurosci..

[26] Hytten F. (1999). Clinical physiology in obstetrics. Eur. J. Obstet. Gynecol. Reprod. Biol..

[27] Hytten F.E., Thomson A.M., Taggart N. (1966). Total body water in normal pregnancy. J. Obstet. Gynaecol. Br. Commonw..

[28] Beall M.H., van den Wijngaard J.P., van Gemert M.J., Ross M.G. (2007). Amniotic fluid water dynamics. Placenta.

[29] McKenzie A.L., Perrier E.T., Guelinckx I., Kavouras S.A., Aerni G., Lee E.C., Volek J.S., Maresh C.M., Armstrong L.E. (2017). Relationships between hydration biomarkers and total fluid intake in pregnant and lactating women. Eur. J. Nutr..

[30] Savitz D.A., Andrews K.W., Pastore L.M. (1995). Drinking water and pregnancy outcome in central North Carolina: Source, amount, and trihalomethane levels. Environ. Health Perspect..

[31] Shahnazi M., Tagavi S., Hajizadeh K., Farshbaf Khalili A. (2013). The effects of intravenous hydration on amniotic fluid index in pregnant women with preterm premature rupture of membranes: A randomized clinical trial. J. Caring Sci..

[32] Kilpatrick S.J., Safford K.L. (1993). Maternal hydration increases amniotic fluid index in women with normal amniotic fluid. Obstet. Gynecol..

[33] Doi S., Osada H., Seki K., Sekiya S. (1998). Effect of maternal hydration on oligohydramnios: A comparison of three volume expansion methods. Obstet. Gynecol..

[34] Yasuda R., Takeuchi K., Funakoshi T., Maruo T. (2003). Bioelectrical impedance analysis in the clinical management of preeclamptic women with edema. J. Perinat. Med..

[35] Zhang J., Zhang N., Liang S., Wang Y., Liu S., Liu S., Du S., He H., Xu Y., Cai H. (2019). The amounts and contributions of total drinking fluids and water from food to total water intake of young adults in Baoding, China. Eur. J. Nutr..

[36] Guelinckx I., Tavoularis G., König J., Morin C., Gharbi H., Gandy J. (2016). Contribution of water from food and fluids to total water intake: Analysis of a French and UK population surveys. Nutrients.

[37] Lee K.W., Shin D., Song W.O. (2016). Total water intake from beverages and foods is associated with energy intake and eating behaviors in Korean adults. Nutrients.

[38] Ding Y., Xie Z., Lu X., Luo H., Pan H., Lin X., Wu J., Wang Z. (2021). Water intake in pregnant women in China, 2018: The report of a survey. Nutrients.

[39] Zhou Y., Zhu X., Qin Y., Li Y., Zhang M., Liu W., Huang H., Xu Y. (2019). Association between total water intake and dietary intake of pregnant and breastfeeding women in China: A cross-sectional survey. BMC Pregnancy Childbirth.

[40] Society C.N. (2014). Chinese Dietary Reference Intakes 2013.

[41] Roussel R., Fezeu L., Bouby N., Balkau B., Lantieri O., Alhenc-Gelas F., Marre M., Bankir L., Group D.E.S.I. (2011). Low water intake and risk for new-onset hyperglycemia. Diabetes Care..

[42] Pross N., Demazières A., Girard N., Barnouin R., Metzger D., Klein A., Perrier E., Guelinckx I. (2014). Effects of changes in water intake on mood of high and low drinkers. PLoS ONE.

[43] Association T.W.C.O. (2015). General Standard for Beverage.

[44] Perrier E.T., Buendia-Jimenez I., Vecchio M., Armstrong L.E., Tack I., Klein A. (2015). Twenty-four-hour urine osmolality as a physiological index of adequate water intake. Dis. Markers.

[45] Suh H., Summers L.G., Seal A.D., Colburn A.T., Mauromoustakos A., Perrier E.T., Bottin J.H., Kavouras S.A. (2020). Afternoon urine osmolality is equivalent to 24 h for hydration assessment in healthy children. Eur. J. Clin. Nutr..

[46] Bar-David Y., Urkin J., Landau D., Bar-David Z., Pilpel D. (2009). Voluntary dehydration among elementary school children residing in a hot arid environment. J. Hum. Nutr. Diet..

[47] Ma G. (2019). Hydration status and health. Chin. J. Prev. Med..

[48] Kavouras S.A. (2019). Hydration, dehydration, underhydration, optimal hydration: Are we barking up the wrong tree?. Eur. J. Nutr..

[49] Keilman C., Shanks A.L. (2022). Oligohydramnios.

[50] WHO (2011). Hamoglobin Concentrations for the Diagnosis of Anemia and Assessment of Severity.

[51] Kumar A., Agarwal K., Devi S.G., Gupta R.K., Batra S. (2010). Hypocalcemia in pregnant women. Biol. Trace Elem. Res..

[52] Committee opinion no. (2011). 504: Screening and diagnosis of gestational diabetes mellitus. Obstet. Gynecol..

[53] Claros N.M. (2017). Gestational hypertension. Hipertens. Riesgo Vasc..

[54] Steiner P.E., Lushbaugh C.C. (1986). Landmark article, Oct. 1941: Maternal pulmonary embolism by amniotic fluid as a cause of obstetric shock and unexpected deaths in obstetrics. JAMA-J. Am. Med. Assoc..

[55] Zhao H., Wong R.J., Stevenson D.K. (2021). The impact of hypoxia in early pregnancy on placental cells. Int. J. Mol. Sci..

[56] Sibai B.M. (2005). Diagnosis, prevention, and management of eclampsia. Obstet. Gynecol..

[57] Black R.E., Allen L.H., Bhutta Z.A., Caulfield L.E., de Onis M., Ezzati M., Mathers C., Rivera J., Group M.A.C.U. (2008). Maternal and child undernutrition: Global and regional exposures and health consequences. Lancet.

[58] Chinese N.S. (2022). Dietary Guidelines for Chinese Residents: 2022.

[59] Liyan Z., Sheng G. (2020). Survey on diet and drinking water in early pregnancy and its correlation with body composition. Food Nutr. China.

[60] Pan W., Wu W., Chen Y., Wang Z., Wang D., Cai L. (2020). Chinese diet balance index for pregnancy: Revision and application. Acta Nutr. Sin..

[61] Zhang N., Zhang J., Wang X., Li Y., Yan Y., Ma G. (2022). Behaviors of water intake, hydration status, and related hydration biomarkers among physically active male young adults in Beijing, China: A cross-sectional study. Int. J. Clin. Pract..

[62] Nuccio R.P., Barnes K.A., Carter J.M., Baker L.B. (2017). Fluid balance in team sport athletes and the effect of hypohydration on cognitive, technical, and physical performance. Sports Med..

[63] Zhang J., Zhang N., Wang Y., Liang S., Liu S., Du S., Xu Y., He H., Cai H., Ma G. (2020). Drinking patterns and hydration biomarkers among young adults with different levels of habitual total drinking fluids intake in Baoding, Hebei Province, China: A cross-sectional study. BMC Public Health.

[64] Zhang J., Zhang N., Liu S., Du S., Ma G. (2022). Young adults with higher salt intake have inferior hydration status: A cross-sectional study. Nutrients.

[65] Stookey J.D., Hamer J., Killilea D.W. (2017). Change in hydration indices associated with an increase in total water intake of more than 0.5 L/day, sustained over 4 weeks, in healthy young men with initial total water intake below 2 L/day. Physiol. Rep..

[66] Zhang J., Ma G., Du S., Liu S., Zhang N. (2021). Effects of water restriction and Supplementation on cognitive performances and mood among young adults in Baoding, China: A randomized controlled trial (RCT). Nutrients.

[67] Szinnai G., Schachinger H., Arnaud M.J., Linder L., Keller U. (2005). Effect of water deprivation on cognitive-motor performance in healthy men and women. Am. J. Physiol.-Regul. Integr. Comp. Physiol..

[68] Heen E., Yassin A.A., Madar A.A., Romøren M. (2021). Estimates of fluid intake, urine output and hydration-levels in women from Somaliland: A cross-sectional study. J. Nutr. Sci..

[69] Armstrong L.E., Johnson E.C., Munoz C.X., Swokla B., Le Bellego L., Jimenez L., Casa D.J., Maresh C.M. (2012). Hydration biomarkers and dietary fluid consumption of women. J. Acad. Nutr. Diet..

